# The Gut Microbiota in Inflammatory Bowel Disease

**DOI:** 10.3389/fcimb.2022.733992

**Published:** 2022-02-22

**Authors:** Peng Qiu, Takatsugu Ishimoto, Lingfeng Fu, Jun Zhang, Zhenyong Zhang, Yang Liu

**Affiliations:** ^1^ Department of Anesthesiology, Shengjing Hospital of China Medical University, Shenyang, China; ^2^ Department of Gastroenterological Surgery, Graduate School of Medical Sciences, Kumamoto University, Kumamoto, Japan; ^3^ Gastrointestinal Cancer Biology, International Research Center for Medical Sciences, Kumamoto University, Kumamoto, Japan; ^4^ Department of Oncology, Shengjing Hospital of China Medical University, Shenyang, China

**Keywords:** gut microbiota, inflammatory bowel disease, treatment, metabolite, IBD

## Abstract

Epidemiological surveys indicate that the incidence of inflammatory bowel disease (IBD) is increasing rapidly with the continuous growth of the economy. A large number of studies have investigated the relationship between the genetic factors related to the susceptibility to IBD and the gut microbiota of patients by using high-throughput sequencing. IBD is considered the outcome of the interaction between host and microorganisms, including intestinal microbial factors, abnormal immune response, and a damaged intestinal mucosal barrier. The imbalance of microbial homeostasis leads to the colonization and invasion of opportunistic pathogens in the gut, which increases the risk of the host immune response and promotes the development of IBD. It is critical to identify the specific pathogens related to the pathogenesis of IBD. An in-depth understanding of various pathogenic factors is of great significance for the early detection of IBD. This review highlights the role of gut microbiota in the pathogenesis of IBD and provides a theoretical basis for the personalized approaches that modulate the gut microbiota to treat IBD.

## Introduction

Inflammatory bowel disease (IBD) consists of 2 subtypes: Crohn’s disease (CD) and ulcerative colitis (UC), which affects 0.3%–0.5% of the global population (Ng et al., 2017). The occurrence and development of IBD, which is a type of idiopathic inflammatory gastrointestinal disease, are influenced by multiple etiologies, including genetic susceptibility, immune factors, and the gut microbiota (Manichanh et al., 2012; Schaubeck et al., 2016). Several studies have confirmed that the composition of gut microbiota in IBD patients is significantly different from that of healthy individuals (Mosca et al., 2016; Oligschlaeger et al., 2019).

The human microbiota comprises 10–100 trillion microorganisms (Mukhopadhya et al., 2015; Lopetuso et al., 2016; Marchesi et al., 2016), including bacteria, viruses, protozoa, and fungi, among which bacteria are the most abundant, with a density of 10^11^–10^12^ cells/ml. More than 99% of the bacteria belong to the phyla *Firmicutes*, *Bacteroides*, *Proteus*, and *Actinomycetes*, whereas *Firmicutes* and *Bacteroides* are dominant in the gut flora of the healthy host (Kostic et al., 2014; Lloyd-Price et al., 2019). Over 1,000 species of bacteria in the gastrointestinal tract play a fundamental role in several aspects of host homeostasis: nutrition, immune, metabolism, and defense against pathogens (Wilson and Nicholson, 2017). The gut microbiota can decompose carbohydrates and indigestible oligosaccharides in food, synthesize short-chain fatty acids (SCFAs), such as butyric acid, propionic acid, and acetate, and provide abundant energy for the intestinal epithelium (Ramakrishna, 2013). Beneficial bacteria in the gut microbiota can play an immunosuppressive role by regulating host immune cells (Allen-Vercoe and Coburn, 2020). Some harmful bacteria can also induce inflammatory cytokines by immune cell interactions or their metabolites to promote the intestinal damage (Stappenbeck and Virgin, 2016). Herein, we review the pathogenic interaction of the microbial communities with the intestinal epithelial barrier, metabolome, and immune system of patients with IBD, and discuss the practical strategies used by microbiota-based therapies to treat IBD patients.

## Microbiota Composition and IBD

The composition and diversity of the gut microbiota are key factors leading to the development of IBD (Lavelle et al., 2019; Schirmer et al., 2019). The composition of the gut microbiota can change in the early stages of IBD. The fluctuation of gut microbiota composition in IBD patients is greater than that in healthy individuals (Halfvarson et al., 2017). Some studies have found that the degree of dysbiosis in CD patients is greater than that in individuals with UC (Cekin, 2017; Kriss et al., 2018; Yilmaz et al., 2018). Compared with healthy controls, the levels of *Bifidobacterium longum* in UC, *Eubacterium rectale*, *Faecalibacterium prausnitzii*, *Roseburia intestinalis*, and other beneficial bacteria in CD and UC were significantly reduced, while the relative abundance and growth rate of harmful bacteria such as *Bacteroides fragilis* are increased (Vich Vila et al., 2018). *Ruminococcus torques* and *Ruminococcus* are also enriched in CD and UC at the onset of the disease. The transcriptional activity of a small number of strains increases as well, as shown by the significant differences in the abundance of *Clostridium hathewayi*, *Clostridium bolteae*, and *Ruminococcus gnavus* (Lloyd-Price et al., 2019). The families of *Christensenellaceae*, *Coriobacteriaceae*, and, in particular, *Clostridium leptum* decrease, while *Actinomyces* spp., *Veillonella* spp., and also *Escherichia coli* increase in patients with CD. For patients with UC, there is an enrichment of *Eubacterium rectum* and *Akkermansia muciniphila* decreases, while levels of *E. coli* increase (Pittayanon et al., 2020). A comparative study showed that the abundance of *Intestinibacter* spp. increases in both CD and UC, while the abundance of *Coprococcus* spp. significantly decreases in CD (Forbes et al., 2018). Hall et al. found that *R. gnavus* is significantly more abundant in patients with IBD.

**Figure 1:**
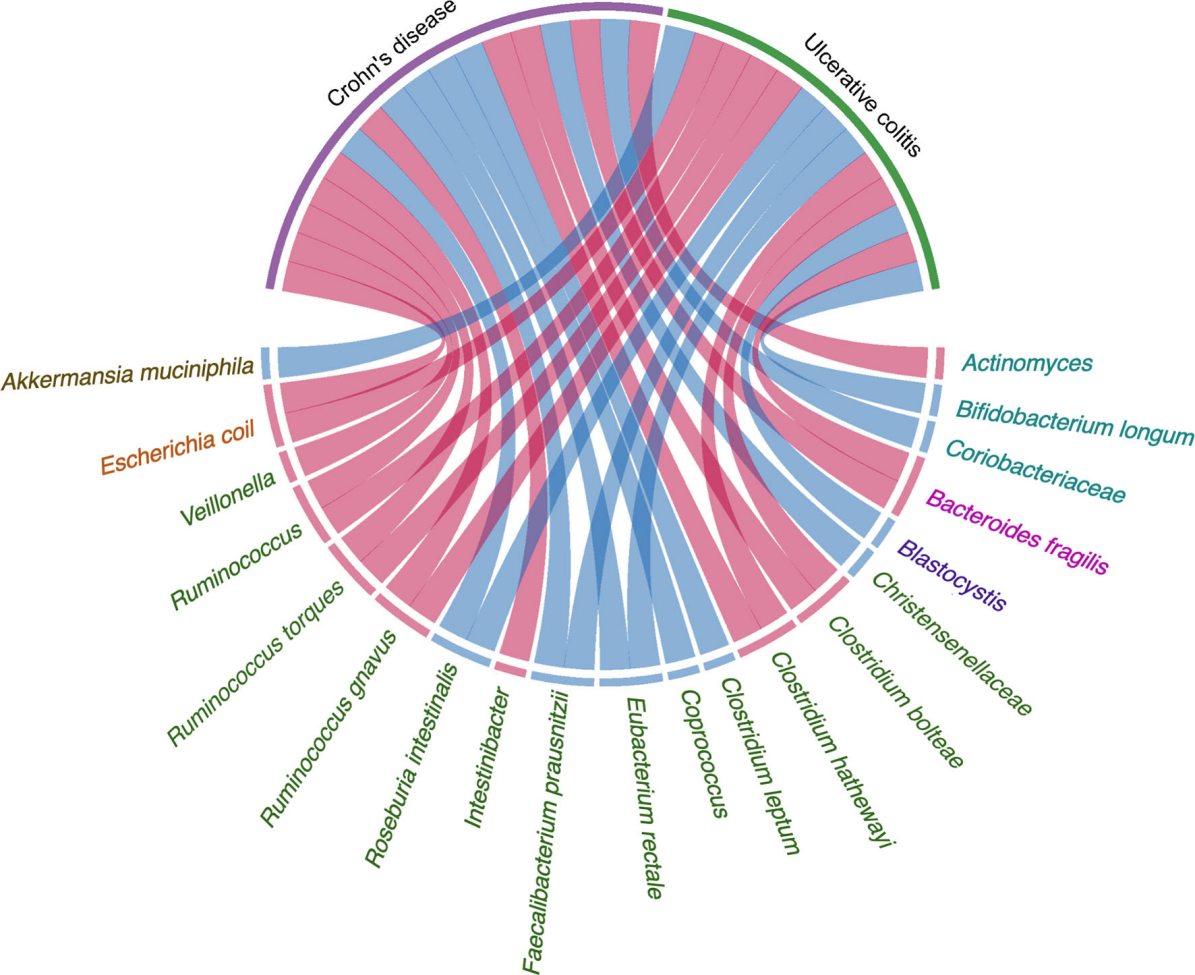
Evidence of the gut microbiota enriched in UC and CD playing a vital role in pathogenesis. Circos plots showing the correlation of bacteria with pathogenesis in IBD. The red ribbons represent the higher production of bacteria enriched in IBD development. The blue ribbons represent the lower production of bacteria enriched in IBD. The causality of the microbiota in IBD has not yet been fully elucidated. Different taxa are divided into six groups and colored by the phylum.

A total of 199 IBD-specific genes have been identified that are involved in adhesion, oxidative stress responses, and utilization of the mucus, which favor the colonization of *R. gnavus* in IBD (Hall et al., 2017). *A. muciniphila* was demonstrated to be a pathobiont that promotes the development of IBD and NOD-like receptor 6 (NLRP6) and was identified as a key regulator of the abundance of *A. muciniphila* (Seregin et al., 2017). IBD-related genes Caspase recruitment domain family member 9 (CARD9), Nucleotide binding oligomerization domain containing 2 (NOD2), Autophagy related 16 like 1(ATG16L), Immunity related GTPase M (IRGM), and Fucosyltransferase 2 (FUT2) are significantly associated with the low abundance in the genus *Roseburia* (Imhann et al., 2018). The prevalence of *Blastocystis* spp. was reduced in patients with active IBD compared with healthy individuals (Tito et al., 2019). Weersma et al. used 12 exome-wide microbial quantitative trait loci (mbQTL) analyses, identified variants in several genes, including Myelin gene regulatory factor (MYRF), SEC16 homolog A (SEC16A), Interleukin 17 receptor E-like (IL17REL), and WD repeat domain 78 (WDR78), which were related with IBD. The genetic variants affecting the immune system play a vital role in shaping the microbiota in the etiology of IBD (Hu et al., 2021). There is increasing evidence that the influence of genetic susceptibility and the environment on gut microbiota is also related to the pathogenesis of IBD (**Figure 1** and **Table 1**).

**Table 1:** The classification and abundance of gut microbiota and microbiota-derived metabolites in IBD.

IBD subtypes	Gut microbiota or metabolite	Abundance compared with healthy people	Ref.
UC	*Bifidobacterium longum*	Low	(Vich Vila et al., 2018)
UC & CD	*Eubacterium rectale*	Low	(Vich Vila et al., 2018)
UC & CD	*Faecalibacterium prausnitzii*	Low	(Vich Vila et al., 2018)
UC & CD	*Roseburia intestinalis*	Low	(Vich Vila et al., 2018)
	*Bacteroides fragilis*	High	(Vich Vila et al., 2018)
UC & CD	*Ruminococcus torques*	High	(Lloyd-Price et al., 2019)
UC & CD	*Ruminococcus*	High	(Lloyd-Price et al., 2019)
UC & CD	*Clostridium hathewayi*	High	(Lloyd-Price et al., 2019)
UC & CD	*Clostridium bolteae*	High	(Lloyd-Price et al., 2019)
UC & CD	*Ruminococcus gnavus*	High	(Hall et al., 2017; Lloyd-Price et al., 2019)
CD	Christensenellaceae	Low	(Pittayanon et al., 2020)
CD	Coriobacteriaceae	Low	(Pittayanon et al., 2020)
CD	*Clostridium leptum*	Low	(Pittayanon et al., 2020)
CD	*Actinomyces*	High	(Pittayanon et al., 2020)
CD	*Veillonella*	High	(Pittayanon et al., 2020)
UC & CD	*Escherichia coli*	High	(Pittayanon et al., 2020)
UC	*Eubacterium rectum*	Low	(Pittayanon et al., 2020)
UC	*Akkermansia muciniphila*	Low	(Pittayanon et al., 2020)
UC & CD	*Intestinibacter*	High	(Forbes et al., 2018)
CD	*Coprococcus*	Low	(Forbes et al., 2018)
UC & CD	*Blastocystis*	Low	(Tito et al., 2019)
UC & CD	Sphingolipids	High	(Franzosa et al., 2019)
UC & CD	Bile acid	High	(Hang et al., 2019)
UC & CD	Triacylglycerol	Low	(Franzosa et al., 2019)
UC & CD	Tetrapyrrole	Low	(Franzosa et al., 2019)
UC & CD	SCFAs	Low	(Chattopadhyay et al., 2021)
UC & CD	Tryptophan	High	(Nikolaus et al., 2017)
UC & CD	N-acylethanolamine	High	(Fornelos et al., 2020)

IBD, Inflammatory bowel disease; CD, Crohn’s disease; UC, ulcerative colitis.

## Microbiota Interact With the Intestinal Epithelial Barrier in IBD

The intestinal barrier includes mechanical, chemical, immune, and microbial barriers. Roy et al. demonstrated that a distinct intestinal microbial community associated with the development of IBD is likely through its associated damage to the intestinal barrier *via* immune cells (Roy et al., 2017). Increasing evidence demonstrates that glycosylation of intestinal epithelial cells leads to increased expression of truncated O-glycans with altered expression of terminal glycan structures. Variability in glycan composition can disrupt the mucosal layer and immunity and ultimately contribute to the onset of IBD (Kudelka et al., 2020). Increased intestinal permeability has been proven to play a role in the pathogenesis of IBD (Llewellyn et al., 2018). The destruction of tight junction proteins leads to the damage of the intestinal mechanical barriers (Coyne and Comstock, 2019). A typical feature of IBD is dysbiosis of the gut microbiota, which results in the imbalance between beneficial and harmful bacteria taxa, and results in damage to the intestinal microbial barrier (Chelakkot et al., 2018). Some studies have linked the gut microbiota to metabolic defects of intestinal epithelial cells, especially through the Nod-like receptor (NLR) family. NLRX1 (nucleotide-binding oligomeric domain, X1 rich leucine-rich repeat) is a mitochondria-related NLR, which has a potential anti-inflammatory effect on colitis. NLRX1 is essential to maintain a balanced glutamine metabolism and barrier function in intestinal epithelial cells. The abundance of colitis-associated pathogens such as *Veillonella* spp. and *Clostridium* spp. in NLRX1 knockout mice increased. Supplementary feeding of glutamine can alleviate inflammation and induce changes in the gastrointestinal flora in NLRX1 knockout mice, while NLRX1 deletion affects the SIRT1 signaling pathway (Leber et al., 2018). Paneth cells are activated in SIRT1 knockout mice and promote nuclear factor kappa-B (NF-κB) pathway activation and ileum inflammation. The fecal microbiota is altered in SIRT1 knockout mice due to changes in bile acid metabolism. Moreover, SIRT1 knockout mice with gut microbiota dysbiosis have developed more serious colitis than control mice (Wellman et al., 2017). Previous studies have also shown a link between mitochondrial dysfunction and IBD (Rath et al., 2018; Mancini et al., 2021). In addition, mitochondrial dysfunction in intestinal epithelial cells and Paneth cells can induce ileal wall inflammation in mice. Mitochondrial respiratory dysfunction forces intestinal epithelial cells to acquire the abnormal phenotype of Paneth cells, resulting in metabolic imbalance and inflammation (Khaloian et al., 2020). Mitochondrial damage in patients with CD is also related to the decrease of H_2_S detoxification and the increase in the relative abundance of H_2_S-producing bacteria. The abundance of *Atopobium parvulum*, a type of H_2_S-producing bacteria, is related to the severity of CD patients. In CD, over 50% of patients present adherent-invasive *E. coli* (AIEC) colonization in the intestinal mucosa (Shawki and McCole, 2017). AIEC penetrate the mucus layer and adhere to intestinal epithelial cells through FimH and cell adhesion molecule 6 (Ceacam 6), and then colonize in the intestinal mucosa (Palmela et al., 2018). *Enterobacteriaceae* can use soluble factors released by apoptotic intestinal epithelial cells to promote the growth and colonization by driving the pyruvate formate-lyase-encoding pflB gene to induce the deterioration of IBD (Anderson et al., 2021). *Klebsiella pneumoniae* invades intestinal epithelial cells and interacts with macrophages to drive the release of interleukin (IL)-1β and tumor necrosis factor (TNF) (Read et al., 2021). *Fusobacterium nucleatum* can upregulate caspase recruitment domain 3 (CARD3) by Nucleotide-binding oligomerization domains 2 (NOD2) in colonic epithelial cells and thereby activates IL-17F/NF-κB signaling pathway and promotes the occurrence of intestinal inflammation (Chen et al., 2020).

The gut microbiota can produce various metabolites to prevent the invasion of pathogenic bacteria and promote intestinal homeostasis (Deleu et al., 2021). The gut microbiota and mucus secreted by Paneth cells play a vital role in the chemical barrier of the intestinal tract. SCFAs are an important metabolite produced by intestinal flora during dietary fiber fermentation (Louis et al., 2014; Lee and Chang, 2021). In addition, gut microbiota will compete with the pathogenic bacteria for nutrition, and the normal flora will play an antagonistic role against the pathogenic bacteria*. F. prausnitzii* can produce butyrate, which plays an anti-inflammatory role by inhibiting the IL-6/signal transducer and activator of transcription 3 (STAT3)/IL-17 pathway and promoting forkhead box protein P3 (Foxp3) (Zhou et al., 2018). Bacteriocin is an important antimicrobial agent. The bacteriocin-producing bacteria inhibits or competes with bacteria of the same species or related species. For example, *Lactobacillus* in humans produce lactobacillin and inhibit the infection of *Listeria monocytogenes* (Rolhion et al., 2019). Enterotoxin secreted by increased Enterotoxigenic *E. coli* (ETEC) increased the permeability of intestinal epithelium, which inhibits the uptake of ascorbic acid *via* the NF-κB pathway (Subramenium et al., 2019).

Immunoglobulin A (IgA) is the most common antibody subtype in the intestine. It is transported by polyclonal immunoglobulin receptor (pIgR) on host epithelial cells and is then released into the intestinal cavity in the form of secretory IgA (sIgA). In a stable environment, sIgA is important for the dynamic balance within the gut microbiota. When the pathogen secretes immunosuppressive protein, it can lead to the destruction of the intestinal immune barrier. IgA-coated bacteria from IBD patients are shown to invade into the mucus layer and exacerbate dextran sulfate sodium (DSS)-induced colitis in mouse models including *Prevotellaceae*, *Helicobacter*, and *Segmented filamentous bacteria* (SFB) (Kayama et al., 2020). The IgA coating patterns of gut microbiota in the IBD cohort were analyzed by using IgA-SEQ profiling, which found that the IgA coating levels of 43 bacterial taxa in IBD patients were higher than those in healthy individuals. The IgA coating levels of *Vibrio* can predict disease progression. Shapiro et al. showed that the analysis of the IgA response to microbiota could be used as a biomarker in the treatment of IBD (Shapiro et al., 2021). As IgA can bind to intestinal bacteria, breastfeeding can reduce the incidence rate of newborn enterocolitis since the specific component IgA in breast milk may mediate this protective effect (Gopalakrishna et al., 2019). Early intervention with FMT could stimulate sIgA secretion and modulate the gut microbiota composition (Cheng et al., 2019).

**Figure 2:**
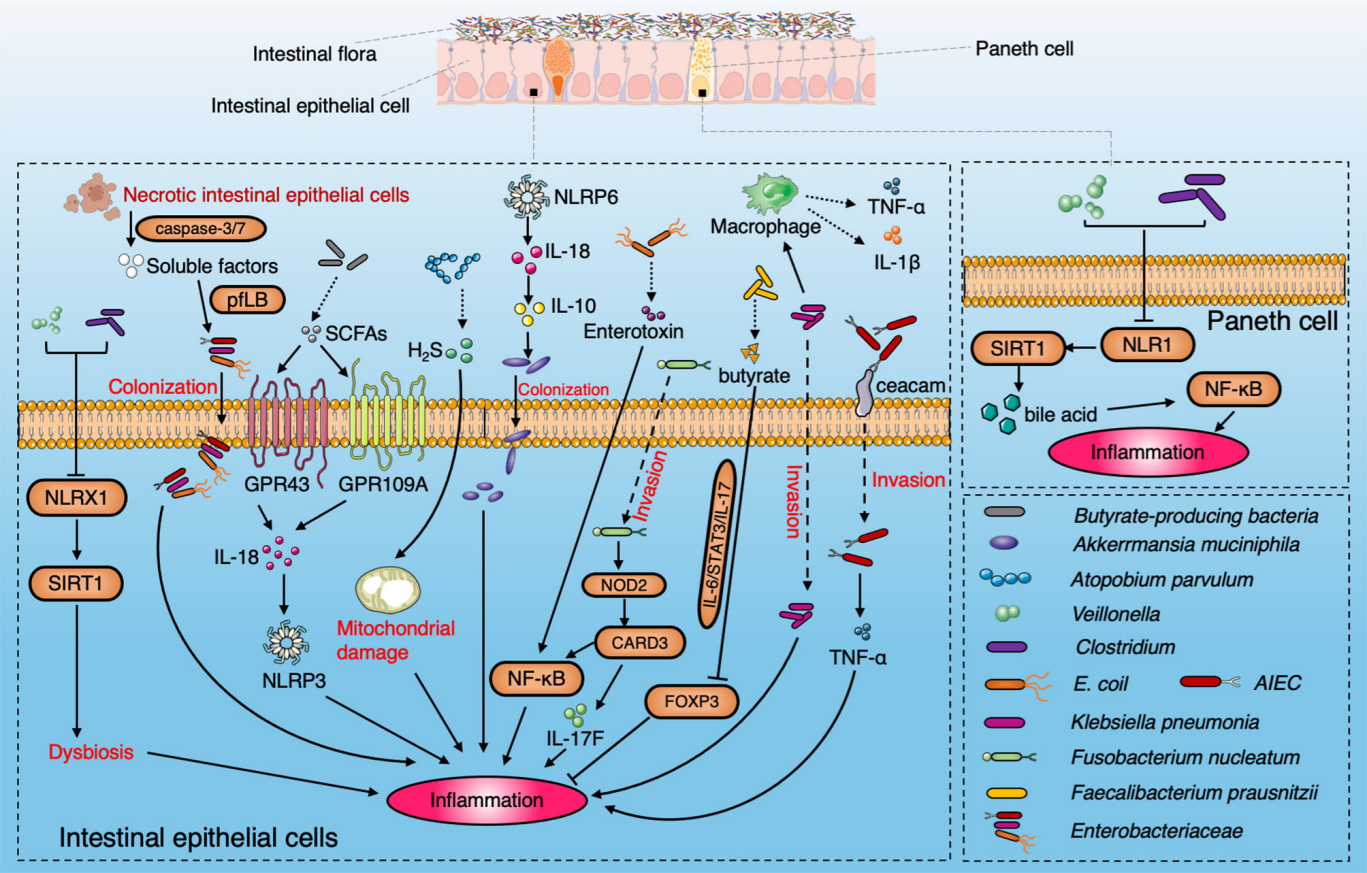
Diagram summarizing the pathogenic interaction between the gut microbiome and intestinal epithelial barrier in IBD. NLRX1 boosts the dysbiosis to induce the ileac inflammation *via* SIRT1 signaling pathway with enrichment of *Veillonella* and *Clostridium*. SCFAs produced by microbiota induce the activation of NLRP3 through GPR43 and GPR109A and protect colitis through IL-18. *A. parvulum* produce H2S to induce ileac inflammation by mitochondrial damage. NLRP6 is a key regulator to facilitate the colonization of *A. muciniphila via* IL-18 and IL-10 to promote IBD onset. AIEC penetrate the mucus layer and adhere to intestinal epithelial cells through FimH and ceacam6. Enterotoxin secreted by *E. coli* induce IBD by the NF-κB pathway. SIRT1 participates in the inflammation by stimulating Paneth cell to reflect the bile acids metabolism *via* the NF-κB signaling pathway. *Klebsiella pneumoniae* invades intestinal epithelial cells and promotes the secretion of IL-1β and TNF by interacting with macrophages. *Fusobacterium nucleatum* upregulates CARD3 *via* NOD2 in colonic epithelial cells to activate the IL-17F/NF-κB signaling pathway. *Faecalibacterium prausnitzii* produce butyrate to inhibit the IL-6/STAT3/IL-17 pathway to activate Foxp3.The soluble factors released by apoptotic intestinal epithelial cells through caspase3/7 facilitate the colonization of *Enterobacteriaceae* by driving the pyruvate formate-lyase-encoding pflB gene.

In addition to the excessive inflammatory response, there are other factors, such as flora imbalance and intestinal barrier disorder, involved in IBD development. Treatment aimed at ameliorating the intestinal barrier and gut microbiota may represent an important objective for the treatment of IBD in the future (**Figure 2**).

## Microbiota-Derived Metabolites in IBD

The intestinal metabolome of IBD patients is disordered and is characterized by an imbalance of SCFA, bile acids, and tryptophan. Xavier et al. identified more than 2,700 bacteria metabolites with altered abundance in IBD. In addition, the production of sphingolipids and bile acid were upregulated in IBD patients, while triacylglycerol and tetrapyrrole were reduced. Overall, more than 50% of previously uncharacterized metabolites were detected, some of which might derive directly from the gut flora (Franzosa et al., 2019). SCFAs regulate mucosal immunity by promoting the development of B cells and the differentiation and expansion of regulatory T cells (Treg), and may activate the production of inflammatory cytokines. Butyric acid can act on immune cells in the intestinal mucosa and may increase the amount and activity of Treg, and inhibit the activity of neutrophils, macrophages, and dendritic cells. The dysbacteriosis and the increase of intestinal inflammatory cells in IBD patients are related to the decrease in SCFAs (Goncalves et al., 2018). The presence of butyrate-producing bacteria, such as *E. coli*, is consistent with the decrease in fecal SCFA levels (Chattopadhyay et al., 2021). SCFAs induce the activation of the NOD-like receptor family, containing three pyridine domains (NLRP3) through its G-protein-coupled receptor 43 (GPR43) and G protein-coupled receptor 109 (GPR109A), which induce ion efflux (K^+^ and Ca^2+^) and promote epithelial repair in colitis by regulating interleukin (IL)-18 (Macia et al., 2015). The effects of SCFA on macrophage polarization is associated with IBD. For example, antibiotic-induced SCFA depletion facilitates the transition to the M1 hyper-reactive phenotype, leading to the production of pro-inflammatory cytokines and promoting intestinal inflammation (Michaudel and Sokol, 2020).

Intestinal bacteria modulate bile acid levels to regulate host immunity (Campbell et al., 2020). Modified bile acids then activate two types of immune cells: Tregs and effector helper T cells, especially Th17, which regulate the immune response by inhibiting or promoting inflammation (Hang et al., 2019). Kasper et al. showed that the gut microbiota could modify bile acids and influence the levels of Treg cells in the mice colon (Song et al., 2020). These researchers speculated that bile acids played an immunomodulatory role by activating Farnesoid X receptor (FXR) and other receptors and decreased the activity of bile salt hydrolase in IBD patients, resulting in an imbalance between primary and secondary bile acids (Gadaleta et al., 2011). Heineken et al. analyzed 693 human gut microbiota genomes and revealed that each bacterium could produce 6 of 13 types of secondary bile acids, while the pairing of two microorganisms could produce 12 kinds of secondary bile acids, suggesting that the biotransformation of bile acids was achieved through interaction with bacteria. The bile acid production of the gut microbiota was significantly reduced in IBD children (Heinken et al., 2019).

Tryptophan is an essential aromatic amino acid. Dietary tryptophan is metabolized by host pathways (casein and serotonin pathway) and microbial pathways (indole pathway). Tryptophan can be metabolized to biologically activate indole by bacteria, which activates aryl hydrocarbon receptors and inhibits the production of inflammatory cytokines (Alexeev et al., 2018; Langan et al., 2021). In the cohort of 535 patients with IBD, the tryptophan metabolism level was associated with the severity of the disease (Nikolaus et al., 2017). In addition, CARD9 promotes the recovery of colitis by activating the IL-22 pathway. The microbiota cannot metabolize tryptophan as a ligand of the aromatic hydrocarbon receptor in the CARD9 knockout mouse model (Lamas et al., 2018). The lack of tryptophan in the diet is associated with the deterioration of colitis in a mouse model (Meisel et al., 2017).

N-acylethanolamine, an endogenous signal lipid, is a metabolite found in IBD, which is associated with *Proteus* enrichment and a reduction in *Bacteroides* levels. Targeting N-acylethanolamine may help to ameliorate IBD-related gut microbiota disorders (Fornelos et al., 2020).

The gut microbiota and its metabolites can regulate the innate and adaptive immune responses. The intervention of microbial metabolites may represent a potential approach for IBD treatment (**Table 1**).

## Interaction Between Immune Cells and the Microbiota in IBD

Stronger antibody and T-cell responses to microbial antigens are common in patients with IBD (Moayyedi et al., 2015; Castellanos et al., 2018; Britton et al., 2020). A variety of immune cells and inflammatory factors participate in the initiation of IBD. The cooperation between immune response mediated by T-cell differentiation subsets and the gut microbiota may affect the occurrence of IBD (Inoue et al., 2005). The inflammatory response driven by Th cells protects the host from harmful pathogens, but the over-activation of Th cells is related to the onset and development of intestinal inflammation. It is generally believed that the occurrence of CD is mainly related to Th1 cells and Th17 cell activation, while the occurrence of UC was the result of the interaction between Th1 and Th2 cells (Chao et al., 2014; Lopetuso et al., 2018). Increasing studies have shown that the incidence of IBD is more correlated with Th17 cell activity. The cytokines secreted by Th17 cells are mainly IL-17 and IL-22, which play a vital role in mediating immune damage and autoimmune diseases. IL-22 is considered to play a protective role when acute colitis occurs; however, it is also found that IL-22 cooperates with IL17A to mediate pathogenicity in chronic colitis (Powell et al., 2020). Necrotic intestinal mucosal cells activate macrophages to produce IL-6 and Transforming growth factor-β (TGF-β) through Signal transducer and activator of transcription 3 (STAT3) and Retinoid related orphan receptors γ (RORγt), which induce the differentiation of Th17 cells (Owaga et al., 2015). Treg cells are mainly used to maintain the balance between T cells. In line with human data, the occurrence of IBD is related to the decrease or abnormal function of Treg cells. Due to the lack of immunosuppressive regulation of Treg cells, effector T cells can trigger an exaggerated immune response in the intestine that will eventually lead to intestinal mucosal injury (Sun et al., 2017). Th17 and Tregs are in equilibrium under normal conditions. The excessive increase of Th17 and the decrease of Tregs cells lead to a disorder in the Th17/Tregs balance. The destruction of this balance can lead to intestinal mucosal damage. IL-6 and low levels of TGF-β can stimulate T cells to differentiate into Th17. High levels of TGF-β can inhibit the production of Th17 cells and promote the production of Treg cells. Th17 cells can also inhibit the proliferation of Treg cells. Th17 cells increase and Tregs decrease in the peripheral blood of IBD patients, indicating that the imbalance of Th17/Tregs plays an important role in the development and maintenance of IBD. The gut microbiota can induce T cells to differentiate into Th17, Treg, and other phenotypes by shaping the intestinal microenvironment. SFB colonized in mouse small intestine can induce Th17 cells in the intestinal lamina propria to secrete IL-17 and IL-22 and promote intestinal inflammation (Lin and Zhang, 2017). Conversely, intestinal bacteria can also promote an increase in Treg cells with anti-inflammatory activity. Honda et al. found that the consumption of *Clostridium* spp. resulted in a significant reduction in intestinal Tregs in germ-free mice, and *Clostridium* spp. colonization promoted the aggregation of RORγ T+FOXP3T+pTreg cells, which, conversely, resulted in inhibiting the response of colonic Th2 and Th17 cells (Ohnmacht et al., 2015). *B. fragilis*, which is symbiotic with human beings, transmits immune regulatory molecules to immune cells through the secretion of outer membrane vesicles (OMVs). This mechanism of OMVs is closely associated with the expression of the IBD-related genes Atg16L1 and NOD2. OMVs play a protective role in IBD by activating the non-classical autophagy pathway. Atg16L1 deficient dendritic cells are unable to induce Tregs to inhibit mucosal inflammation. Human immune cells with Atg16L1 mutation present deficiencies in the Tregs response to OMV (Chu et al., 2016). *B. thetaiotaomicron* recapitulate the effects of gut microbiota and induces Tregs to influence the immune system in IBD (Hoffmann et al., 2016). *F. prausnitzii*-specific circulating CD4 and CD8a (DP8α) T cells have been identified in human colonic mucosa and serum, which exhibit similar features of Treg cells (Godefroy et al., 2018). The ectopic colonization of *K. pneumoniae* in the intestine did not improve the production of anti-inflammatory and regulatory T cells, but preferentially promoted the induction of Th1, showing its unique mechanism of inducing colitis (Atarashi et al., 2017).

**Figure 3:**
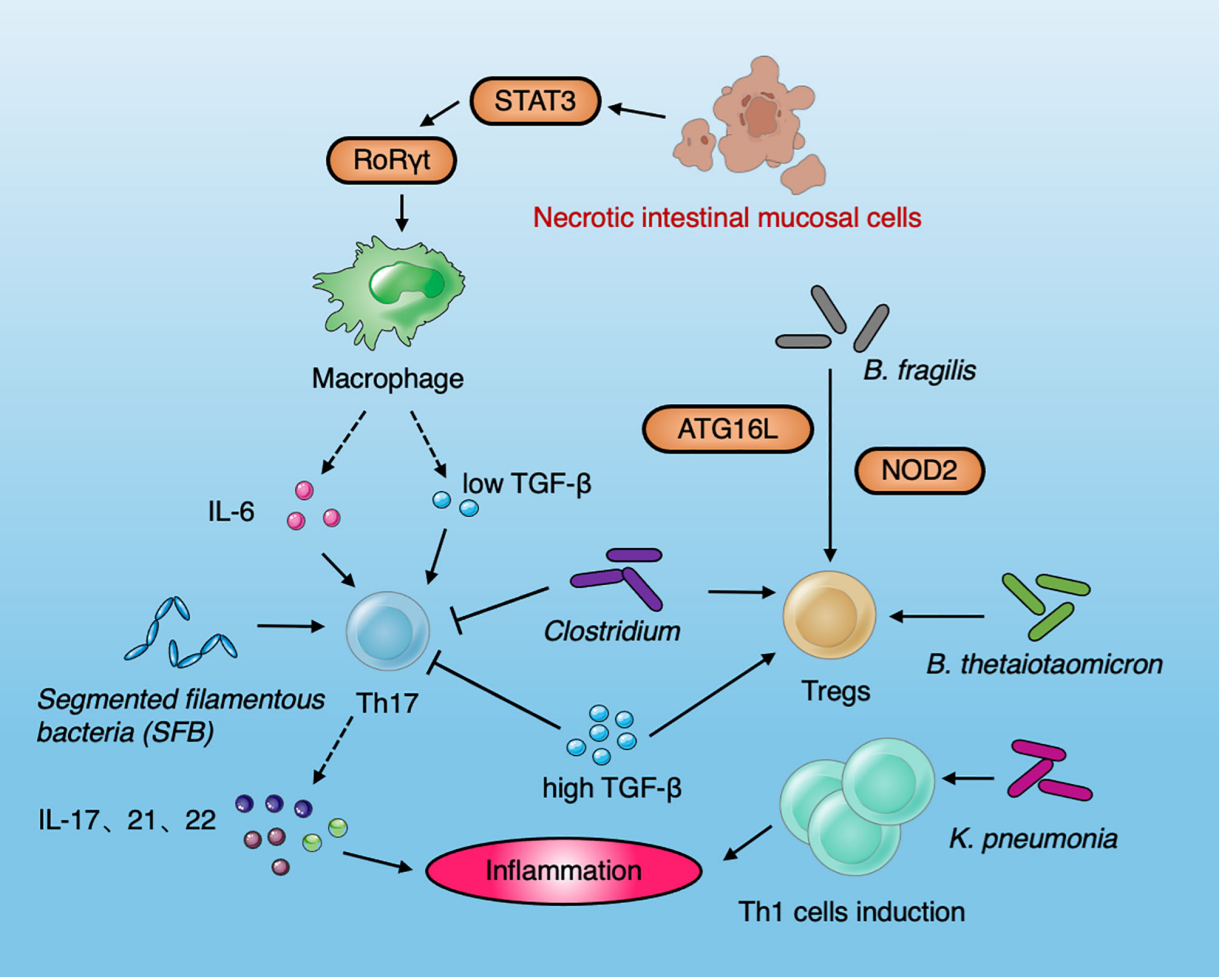
Diagram summarizing the interaction between immune cells and the microbiota in IBD. Necrotic intestinal mucosal cells activate macrophages to produce IL-6 and TGF-β through STAT3 and RORγt, which induce the differentiation of Th17 cells. IL-6 and low levels of TGF-β can stimulate T cells to differentiate into Th 17. High-levels of TGF-β can inhibit the production of Th 17 cells and promote the production of Treg cells. SFB can induce Th17 cells to secrete IL-17 and IL-22 and promote intestinal inflammation. The *Clostridium* spp. resulted in the production of Tregs. *B. fragilis* induced Tregs by the IBD-related genes Atg16L1 and NOD2. *B. thetaiotaomicron* recapitulate the effects of gut microbiota and induce Tregs to influence the immune system in IBD. *Klebsiella pneumoniae* improve the induction of Th1 cell to induce the occurrence of inflammation.

Collectively, the dysbiosis of the gut microbiota, the imbalance of cytokines and the destruction of the mucosal barrier, contribute to induce mucosal inflammation and IBD development (**Figure 3**). Various susceptible genes and environmental factors can interfere with gut microbiota and the host immune system (Zhang et al., 2017).

## Microbiota-Targeted Treatment in IBD

There is a complex dynamic linkage between dysbiosis and the development of IBD, rather than a simple causal relationship (Sartor and Wu, 2017). Probiotics supplementation (especially butyrate-producing bacteria) and fecal microbiota transplantation (FMT) may be used to treat IBD. *E. coli Nissle* 1917 is a probiotic, which can inhibit the growth of *Salmonella* and other pathogenic bacteria. The H1 flagella of *E. coli Nissle* 1917 forms a tight network structure of the intestinal epithelium through the interaction between individual bacteria and anchoring recognition, which can inhibit the adhesion and invasion of pathogenic bacteria to intestinal epithelial cells. *E. coli Nissle* 1917 gene encodes three kinds of fimbriae, F1A, F1C, and curly fimbriae, which are powerful boosters for their continuous colonization and adhesion to intestinal epithelium. In addition, it secretes bacteriocin by competing with pathogens for nutrients in the intestine and antagonize the adhesion of pathogens to intestinal epithelium (Scaldaferri et al., 2016). A large clinical trial studied the efficacy of *E. coli Nissle* 1917 in maintaining the remission after clinical treatment in patients with UC (*n* = 120) compared with mesalazine treatment (1,500 mg/day). It was shown that *E. coli Nissle* 1917 and mesalazine were equivalent in maintaining the remission of UC (Nishida et al., 2021). IBD patients generally present symptoms of iron deficiency, and the iron-deficient intestinal microenvironment is more conducive to the intestinal colonization and curative effect of *E. coli Nissle* 1917. An iron-rich diet may weaken the efficacy of *E. coli Nissle* 1917, and the combination of treatment and diet will achieve twofold results with half the effort (Sassone-Corsi et al., 2016). The genetically engineered *E. coli Nissle* strain was used as a local probiotic to protect mice from chemically induced colitis and promote the mucosal healing (Praveschotinunt et al., 2019). A retrospective analysis of 200 patients with IBD found that probiotics reduced the incidence of side effects such as steroid medication, hospitalization, and surgery (Dore et al., 2020).

Based on the key role of the gut microbiota in the pathogenesis of IBD, FMT can restore the intestinal mucosal immune homeostasis for patients with IBD, which is also a current research hotspot. FMT can be applied for moderate to severe IBD complicated with recurrent or refractory *Clostridium difficile* infection (Colman and Rubin, 2014; Nishida et al., 2017; Goyal et al., 2018; Saha et al., 2021). Physiological and genetic factors related to donors or recipients will affect the curative effect. In addition, the use of specific flora and the method of administration should be reasonably designed and controlled. The factors to be considered in the selection of the flora include the abundance, classification, and composition of the bacterial community and whether to carry out antibiotic pretreatment. At the same time, drug formulation and administration are long-standing problems of FMT (Ng et al., 2020). It was found that triple antibiotics could cooperate with FMT to alleviate the intestinal ecological imbalance caused by the loss of *Bacteroidetes* species diversity in UC patients (Ishikawa et al., 2018). To date, several clinical trials have shown that intensive dose and multi-donor FMT could induce clinical and endoscopic remission (Moayyedi et al., 2015). However, one study found that the transferred microbes in the recipient who underwent FMT 10 weeks later were far from the donor bacteria, which provide a critical issue that the colonization dynamics of gut microbiota should be tracked and manipulated in the recipient of FMT (Chu et al., 2021). In addition, FMT is currently being investigated in registration and ethical clinical research, and it has not been approved for clinical application. Further research is needed to better understand the effective mechanisms underlying the effects of FMT, and to develop more effective regimens to treat IBD (Costea et al., 2017; Allegretti et al., 2019; Caruso et al., 2020).

Anti-tumor necrosis factor (anti-TNF) is a commonly used biological agent, which can prevent the development of inflammation by inhibiting the activity of TNF. At present, there are five kinds of anti-TNF agents: infliximab, adalimumab, etanercept, certolizumab, and golimumab. A German cohort study showed that anti-TNF treatment rendered fecal flora diversity similar to that of the control group. Fecal metabonomics analysis confirmed that the predicted levels of butyrate were significantly correlated with clinical remission after anti-TNF treatment (Aden et al., 2019). Zhuang et al. showed that the diversity and richness of the fecal microbiota in CD patients increased significantly after infliximab treatment, the number of bacteria-producing SCFAs increased, and the number of pathogenic bacteria decreased. Although anti-TNF therapy has changed the drug regimen for treatment of IBD, up to 30% of patients achieved no clinical benefit after the induction period, and up to 50% of patients have to withdraw from treatment (Zhuang et al., 2020).

The diet plays a crucial role in shaping the composition of the microbiota and can be regulated to control IBD symptoms. The effects of the diet on IBD occurs mainly through three mechanisms. First, some diets can change the composition of the intestinal microecology and its metabolic function, thus indirectly affecting intestinal immune function. Second, some dietary components can directly affect the intestinal mucosal barrier and induce a disorder of intestinal mucosal innate immunity indirectly. Finally, some components of the diet can directly participate in the intestinal immune response (Lewis and Abreu, 2017). Exclusive enteral nutrition (EEN) is a basic and polymerized diet without solid food, which is a dietary intervention widely studied in IBD (Levine et al., 2018). EEN has similar effects as corticosteroids in inducing remission in pediatric patients with CD without the known side effects of corticosteroid therapy (Heerasing et al., 2017; Pigneur et al., 2019). EEN is independent of other environmental factors and can rapidly change the composition of the microbiota and effectively reduce the intestinal inflammation of children with CD (Jongsma et al., 2020). The mechanisms by which EEN induces the remission of CD is unclear, but it may promote the growth of beneficial bacteria or the consumption of pathogens. Importantly, host susceptibility to IBD is highly dependent on the combination of specific fiber or protein components that constitute the microbiota, suggesting the importance of dietary composition in IBD.

Vedolizumab is a humanized monoclonal antibody, which can specifically bind to lymphocyte integrin α4β7, and thus inhibits the migration of lymphocytes from vascular endothelium to intestinal tissue (Fleisher et al., 2018). Xavier et al. found that 85 IBD patients receiving vedolizumab responded with high levels of *Roseburia inulinivorans* and *Burkholderiale*s as the therapeutic effect. The authors identified 13 metabolic pathways that were more active in the intestinal bacteria of CD patients after remission (Stevens et al., 2017).

Gut-103 and Gut-108 are two living biotherapy products, which are mainly used to supplement the function of missing or representative deficiency in the dysbiosis of the gut microbiota of IBD patients. Gut-103, composed of 17 bacterial strains, cooperatively provides protection and continuous supplementation to the IBD inflammatory environment to prevent and treat chronic immune-mediated colitis. Gut-108 is an optimized version of Gut-103, which utilizes 11 human bacteria associated with 17 strains, which allow bacteria to remain in the colon longer than other probiotics that normally survive in the gut (van der Lelie et al., 2021).

HABN, a hyaluronic acid-coated bilirubin nanodrug, can accumulate in the inflamed colonic epithelium of mice with acute colitis after oral administration. The strong antioxidant effect of bilirubin allows HABN to protect the epithelial cells of the colon against apoptosis and promotes epithelial barrier recovery. HABN has been shown to regulate the gut microbiota, improve the richness and diversity of the flora, and increase *A. muciniphila* and *Clostridium* XIVα, which were beneficial to intestinal homeostasis. HABN interacts with pro-inflammatory macrophages through hyaluronic acid-CD44 to regulate the innate immune response and to reduce the release of inflammatory cytokines (Lee et al., 2020).

Recently, new drugs for the treatment of IBD have been developed, including agents targeting SMAD7 (Mongersen), IL-12 and IL-23 (ustekinumab), and Janus kinases (tofacitinib, filgotinib, and upadacitinib) (Monteleone et al., 2015; Feagan et al., 2016; Bradley, 2017; Hanauer et al., 2020; Feagan et al., 2021; Parigi et al., 2021). Since *Enterobacteriaceae* are implicated in a unique metabolic pathway whereby its overgrowth interferes with levels of beneficial bacteria, Winter et al. used the heavy metal tungsten to inhibit the proliferation of *Enterobacteriaceae* during IBD inflammation development (Zhu et al., 2018).

Although some regimens have been shown to have the potential or promising effect on IBD (**Table 2**). It is urgent to develop personalized treatment strategies and to identify which treatment regimens would be most beneficial to IBD patients. Using a combination of *in vivo* real-time molecular endoscopy technology, tissue transcriptome analysis, genetic research, gut microbiota analysis, and immune response analysis, we can comprehensively predict the response of patients to specific drugs.

**Table 2:** The impact mechanism of gut microbiota-targeted treatment in IBD.

Microbiota-targeted treatment	Impact mechanism	Reference
Probiotics (*E. coli Nissle* 1917)	Biofilm forming to inhibit pathogenic bacteria	(Sassone-Corsi et al., 2016; Scaldaferri et al., 2016; Praveschotinunt et al., 2019; Dore et al., 2020; Nishida et al., 2021)
FMT	Restore intestinal mucosal homeostasis	(Colman and Rubin, 2014; Costea et al., 2017; Nishida et al., 2017; Ishikawa et al., 2018; Goyal et al., 2018; Allegretti et al., 2019; Caruso et al., 2020; Ng et al., 2020; Chu et al., 2021; Saha et al., 2021)
Anti-TNF	Inhibiting the inflammation of TNF	(Aden et al., 2019; Zhuang et al., 2020)
Diet (EEN)	Change the composition of the microbiota	(Heerasing et al., 2017; Lewis and Abreu, 2017; Levine et al., 2018; Pigneur et al., 2019; Jongsma et al., 2020)
Vedolizumab	Inhibit the migration of lymphocytes from vascular endothelium to intestine	(Stevens et al., 2017; Fleisher et al., 2018)
Gut-103 and Gut-108	Deficient microbiota supplement	(van der Lelie et al., 2021)
HABN	Protect the epithelial cells of colon	(Lee et al., 2020)
Mongersen	target SMAD7	(Monteleone et al., 2015)
Ustekinumab	Target IL-12 and IL-23	(Feagan et al., 2016; Bradley, 2017)
Tofacitinib, Filgotinib, and Upadacitinib	Target Janus kinases	(Hanauer et al., 2020; Feagan et al., 2021; Parigi et al., 2021)
Tungstate	inhibit the proliferation of Enterobacteriaceae	(Zhu et al., 2018)

FMT, fecal microbiota transplantation; Anti-TNF, anti-tumor necrosis factor; EEN. Exclusive enteral nutrition; HABN, Hyaluronic acid-coated bilirubin nanodrug.

## Future Direction

Metabolites derived from the gut microbiota, especially SCFAs, are the key molecules of communication between the gut microbiota and host (Nikolaus et al., 2017; Osaka et al., 2017; Lavelle and Sokol, 2020). It is challenging to decipher all the effects of microbiota on immune metabolism. Part of this complexity is due to the final effects of microbial products, which may vary depending on the environment or the cell type. Functional metagenomics (e.g., expression of a metagenomic DNA library in bacteria such as *E. coli*), synthetic biology and bioinformatics, and the combination of these technologies in a strategic way have proved to be a very powerful approach, emphasizing the widespread presence of biologically active molecules or gene clusters in the population. These methods, using data and technology from different sources to mine the microbial community for new targeting compounds, provide a promising new direction for identifying bioactive molecules related to IBD. It is worth noting that family members of IBD patients can present changes in the metabolic spectrum similar to IBD characteristics, which may help to identify high-risk patients before clinical symptoms occur. In addition, high-resolution time mapping of the microbiome and metabolite markers may provide new biomarkers for predicting progression in diagnosis and treatment. By detecting 16S rRNA and other practical methods, we can target the gut microbiota and carry out the individualized treatment. Multiomics, such as genomics, transcriptomics, proteomics, immunomics, and microbiomics, can accurately analyze the onset of diseases (Lavelle and Sokol, 2018). New therapeutic drugs are constantly being developed, traditional therapeutic drugs are being optimized, and AI algorithms will be applied for the treatment of IBD (Rodrigues et al., 2018). International multi-center collaborative research and large-scale cohort studies will be implemented. Ultimately, an integrated personalized diseases profile (iPOP) will be developed by analyzing the specific disease context of patients at the molecular, cellular, tissue, organ, and body levels, and integrating all these data will help establish an “individual disease” model of each patient. Therefore, we will be able to develop precise therapeutic strategies for patients having similar individual disease spectrums to treat IBD.

## Conclusion

IBD treatment is a long-term and complex process due to the complicated pathogenesis of IBD. Currently, a comprehensive evaluation system involving clinical, biochemical, endoscopic, and other indicators can direct optimal individualized treatment with the aim of predicting drug response. In the future, the combination of intestinal microbiology, gastroenterology epidemiology with rapid analysis of gut microbiota, metabolites, molecular signaling, and genetic engineering is expected to be the new therapeutic direction for IBD.

## Author Contributions

Conception and design: TI, LF, JZ, and YL. Drafting of the manuscript: PQ and YL. Drawing of figures: YL and PQ. Conceiving and critical revision of the manuscript for important intellectual content: TI, ZZ, and YL. All authors contributed to the article and approved the submitted version.

## Funding

This work is supported by the National Natural Science Foundation of China (Grant No. 82103468).

## Conflict of Interest

The authors declare that the research was conducted in the absence of any commercial or financial relationships that could be construed as a potential conflict of interest.

## Publisher’s Note

All claims expressed in this article are solely those of the authors and do not necessarily represent those of their affiliated organizations, or those of the publisher, the editors and the reviewers. Any product that may be evaluated in this article, or claim that may be made by its manufacturer, is not guaranteed or endorsed by the publisher.

## Glossary

**Table:**

IBD	inflammatory bowel disease
CD	Crohn’s disease
UC	ulcerative colitis
SCFAs	short-chain fatty acids
NLRP6	NOD-like receptor
CARD9	caspase recruitment domain family member 9
NOD2	nucleotide binding oligomerization domain containing 2
ATG16L	autophagy related 16 like 1
IRGM	immunity related GTPase M
FUT2	fucosyltransferase 2
mbQTL	microbial quantitative trait loci
MYRF	myelin gene regulatory factor
SEC16A	SEC16 homolog A
IL17REL	interleukin 17 receptor E like
WDR78	WD repeat domain 78
NLR	Nod-like receptor
NLRX1	nucleotide-binding oligomeric domain, X1 rich leucine-rich repeat
AIEC	adherent-invasive Escherichia coli
ETEC	enterotoxigenic *Escherichia coli*
IgA	immunoglobulin A
NF-κB	nuclear factor kappa-B:
SFB	segmented filamentous bacteria
sIgA	secretory IgA
Ceacam 6	cell adhesion molecule 6
Treg	regulatory T cells
NLRP3	NOD-like receptor family containing three pyridine domains
GPR43	G-protein coupled receptor 43
GPR109A	G protein-coupled receptor 109
FXR	farnesoid X receptor
TGF-β	transforming growth factor-β
STAT3	signal transducer and activator of transcription 3
RORγt	retinoid related orphan receptors
OMVs	outer membrane vesicles
FMT	fecal microbiota transplantation
*E. coli*	*Escherichia coli*
anti-TNF	anti-tumor necrosis factor
EEN	exclusive enteral nutrition
HABN	hyaluronic acid-coated bilirubin nanodrug
iPOP	integrated personalized diseases profile
IL	interleukin
DSS	dextran sulfate sodium
